# Cyclophilin B Interacts with Sodium-Potassium ATPase and Is Required for Pump Activity in Proximal Tubule Cells of the Kidney

**DOI:** 10.1371/journal.pone.0013930

**Published:** 2010-11-10

**Authors:** Guillermo Suñé, Eduard Sarró, Marta Puigmulé, Joan López-Hellín, Madeleine Zufferey, Thomas Pertel, Jeremy Luban, Anna Meseguer

**Affiliations:** 1 Fisiopatología Renal, Centre d'Investigacions en Bioquímica i Biologia Molecular (CIBBIM), Institut de Recerca Vall d'Hebron (VHIR), Hospital Universitari Vall d'Hebron, Barcelona, Spain; 2 Departament de Bioquimica i Biologia Molecular, Facultat de Medicina, Universitat Autónoma de Barcelona, Barcelona, Spain; 3 Department of Microbiology and Molecular Medicine, University of Geneva, Geneva, Switzerland; Universidade de Sao Paulo, Brazil

## Abstract

Cyclophilins (Cyps), the intracellular receptors for Cyclosporine A (CsA), are responsible for peptidyl-prolyl cis-trans isomerisation and for chaperoning several membrane proteins. Those functions are inhibited upon CsA binding. Albeit its great benefits as immunosuppressant, the use of CsA has been limited by undesirable nephrotoxic effects, including sodium retention, hypertension, hyperkalemia, interstial fibrosis and progressive renal failure in transplant recipients. In this report, we focused on the identification of novel CypB-interacting proteins to understand the role of CypB in kidney function and, in turn, to gain further insight into the molecular mechanisms of CsA-induced toxicity. By means of yeast two-hybrid screens with human kidney cDNA, we discovered a novel interaction between CypB and the membrane Na/K-ATPase β1 subunit protein (Na/K-β1) that was confirmed by pull-down, co-immunoprecipitation and confocal microscopy, in proximal tubule-derived HK-2 cells. The Na/K-ATPase pump, a key plasma membrane transporter, is responsible for maintenance of electrical Na+ and K+ gradients across the membrane. We showed that CypB silencing produced similar effects on Na/K-ATPase activity than CsA treatment in HK-2 cells. It was also observed an enrichment of both alpha and beta subunits in the ER, what suggested a possible failure on the maturation and routing of the pump from this compartment towards the plasma membrane. These data indicate that CypB through its interaction with Na/K-β1 might regulate maturation and trafficking of the pump through the secretory pathway, offering new insights into the relationship between cyclophilins and the nephrotoxic effects of CsA.

## Introduction

Cyclosporine A (CsA) is a potent immunosuppressive cyclic undecapeptide mainly used to prevent graft rejection after transplant surgery [1]. It has changed the outcomes of transplantation and autoimmune diseases, although with the drawback of significant nephrotoxicity [2]. CsA nephrotoxic effects include a reduction in glomerular filtration rate by increasing arteriolar vasoconstriction and renal vascular resistance, microvascular arteriolopathy, interstitial fibrosis, and vacuolization of proximal tubule cells [3], [4]. Experimental models of CsA nephrotoxicity in rats, in which glomerular hemodynamics were analyzed by renal micropuncture techniques, revealed that CsA administration is associated with afferent and efferent arteriolar vasoconstriction, with predominating preglomerular vasoconstriction that results in a significant reduction of renal plasma flow. A reduction of the ultrafiltration coefficient has also been observed. The decrease in these two hemodynamic variables lead to a significant reduction of the single-nephron glomerular filtration rate and thus renal dysfunction [3]. Hyperkalemia and metabolic acidosis have been reported as negative side effects, as well. Studies in rats showed a distal tubular acidification defect and a significant reduction in sodium and potassium urinary excretion by intraperitoneal administration of CsA [5], [6]. The molecular mechanisms underlying such effects have not been completely uncovered. Cyclophilins, first discovered as the intracellular receptors of CsA, are conserved and ubiquitous enzymes that exhibit peptidyl prolyl *cis-trans* isomerases (PPIases) activity and are involved in folding and repair of proteins, as well as, acting as chaperones in both prokaryotes and eukaryotes [7]. There are more than ten subtypes of CyPs in mammals, distributed in different subcellular compartments, that function in numerous cellular processes, including transcriptional regulation, immune response, protein secretion, and mitochondrial function [7]–[9]. CypA, the first characterized cytosolic isoform considered as the major target for CsA [10] has been located in the cytosol. CypB, the second identified member of the family, is distinguished from CypA by the presence of an endoplasmic reticulum-directed signal sequence [11]. We hypothesized that identification of Cyp-interacting proteins in kidney could be relevant to understand CsA nephrotoxicity at the molecular level and, eventually, these proteins might become therapeutical targets to avoid toxicity while maintaining the drug's immunosupresive effects in CsA-treated patients. In this work, we describe that CypB interacts with the Na/K-ATPase pump in human kidney and that this cyclophilin is crucial for pump location and activity.

## Results

### Na/K-ATPase beta 1 subunit interacts with Cyclophilin B

**Figure 1:**
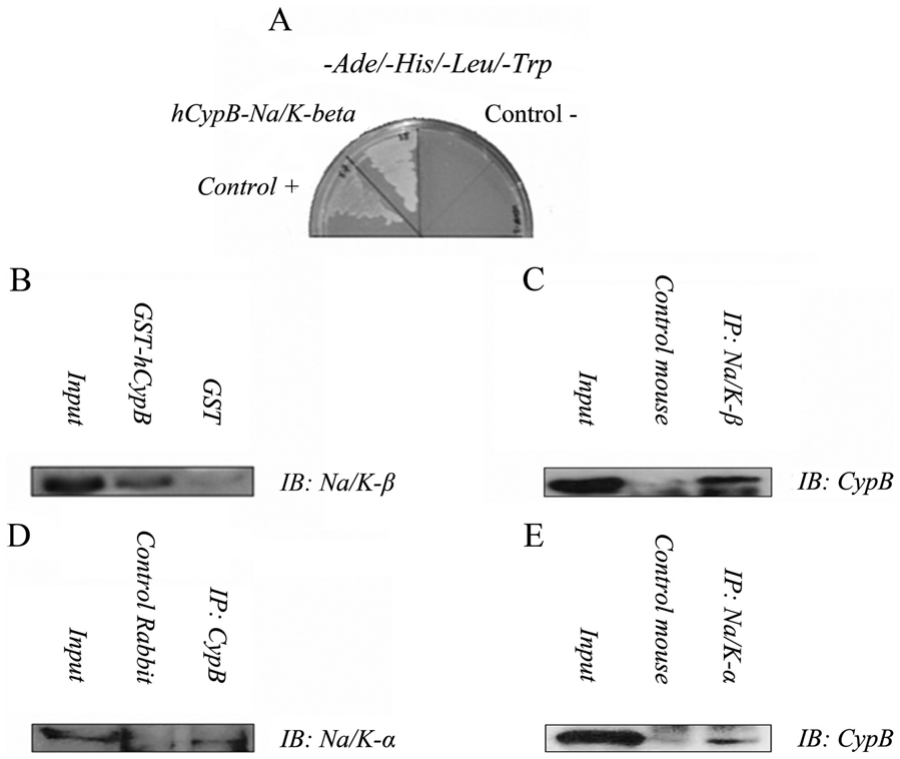
Interaction between CypB and Na/K-ATPase beta and alfa subunits. A: Yeast two hybrid assay. CDNA for CypB was cloned in frame into the yeast expression vector (pGBKT7) that harbours the GAL4 activation domain. The recombinant plasmids plus Human Kidney Matchmaker cDNA Library were co-transformed into AH109 yeast strain. The co-transformed cells were selected on Ade-/Leu-/His-/Trp-, 10 µM Aminotriazol dropout plates to monitor for growth. The positive control represents co-transformation of p53 and T-antigen in two-hybrid expression vectors, pGBKT7-P53 and pGADT7 (BD Biosciences, Clontech), respectively. B: GST-pull-down assays. CypB was fused in frame with the GST gene. GST and GST-HCypB products were immobilized on Sepharose 4B and incubated with COS-7 cells lysates expressing Na/K-β1 (pCMV-HA-Na/K). The bound proteins were analyzed by immunoblotting with anti-Na/K-β1 rabbit polyclonal antibody. C–E: Co-Immunoprecipitation assays. HK-2 lysates were immunoprecipitated with mouse monoclonal antibodies against Na/K-β1 or Na/K-α1 and rabbit polyclonal antibody against CypB. The immunoprecipitates were subjected to Western blotting analyses, as indicated in the figure. As control, ChromePure mouse IgG and ChromePure rabbit IgG were used. Figures 1B to 1E are representative of at least three independent experiments.

The full length human CypB was screened against a human kidney cDNA library in a yeast two-hybrid assay. Nine clones that were effectively interacting with the CypB protein to support the permissive growth of yeast cells in Trp^-^ Ade^-^ His^-^ Leu^-^ selective media, became further selected in 10 µM AT (aminotriazol). One of those clones was identified as the Na/K-ATPase beta subunit 1 (ATP1B1, GenBankTM accession number NM_001677). Fig. 1A shows the interaction between Na/K-β1 and CypB, as well as a positive control for protein interaction involving P53 and SV40 large T antigen proteins (Control +), and a negative control involving the non-interacting lamin C with the SV40 large T antigen (Control -). The plasmid for the Na/K-ATPase beta fusion was isolated from yeast after the primary screen and retested in fresh yeast (not shown).

GST-pull down and co-immunoprecipitation assays were performed to further confirm the CypB-Na/K-β1 interaction. Sepharose 4B conjugated GST and GST-CypB fusion proteins were incubated with COS-7 cell lysates expressing transiently transfected Na/K-β1. After extensive washing in GST buffer, the glutathione beads were boiled in sample buffer, and the supernatant analyzed by SDS-polyacrylamide gel electrophoresis. To recognize the CypB-interacting protein, western blots against Na/K-β1 were performed (Fig. 1B). Results shown in this figure demonstrate that the binding capacity of GST-CypB for Na/K-β1 depends on the CypB moiety since GST alone is unable to bind Na/K-β1. We next performed co-immunoprecipiation assays on HK-2 crude extracts as a demonstration of endogenous interaction and detected CypB in anti-Na/K-β1 immunoprecipitates (Fig. 1C). We also detected the beta-interacting alpha subunit in CypB immunoprecipitates, as expected (Fig. 1D). This was confirmed by reverse co-immunoprecipitation (Fig. 1E). Altogether, these results demonstrated for the first time that CypB interacts with the Na/K-β1, and that the alpha subunit is also co-immunoprecipitated as a part of the complex.

**Figure 2:**
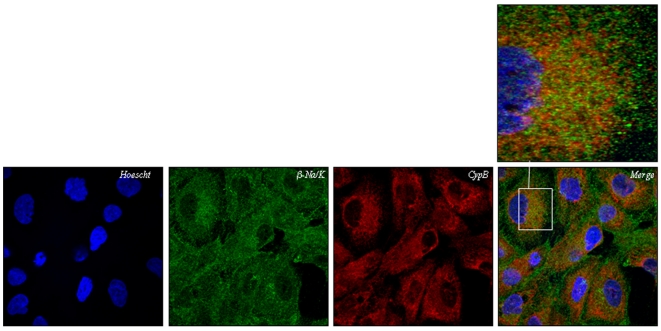
Co-localization experiments between CypB and Na/K-β1 in HK-2 cells. Immunocitochemistry assays using anti-CypB rabbit polyclonal antibody and mouse monoclonal anti-Na/K-β1 antibodies were performed on HK-2 cells. Fluorescence labelling was visualized in a Leica DM IRBE confocal microscope. See inset magnification for co-localization. Figures are representative of at least three independent experiments.

The distribution pattern of endogenous CypB and Na/K-β1 in human kidney derived tubular cells (HK-2) was studied by confocal microscopy. We found that Na/K-β1 location was compatible with the endoplasmic reticulum (ER) and the plasma membrane (Fig. 2). Our results indicate that CypB and Na/K-β1 mainly co-locate in the ER (See inset for enlargement). Such an interaction between the Na/K-β1 and CypB may occur transiently or only along certain steps of the sorting process according to data presented in Fig. 2.

### Effects of CsA treatment on activity, expression and location of Na/K-β1 pump in HK-2 cells

**Figure 3:**
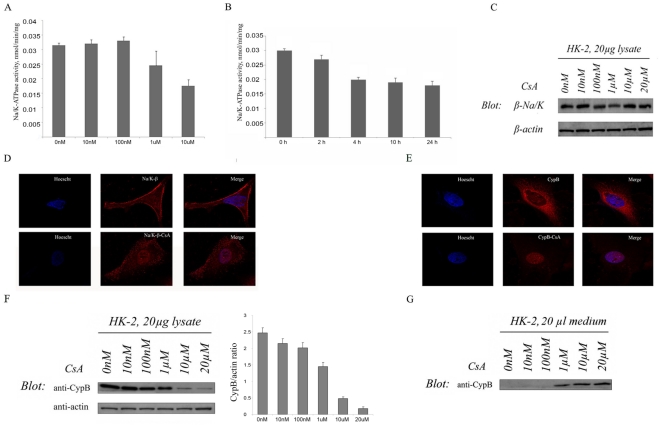
Effects of CsA on Na/K-ATPase in HK-2 cells. A–B. Dose- and time-response effects of CsA on Na/K-ATPase activity. CsA inhibited Na/K-ATPase activity at concentrations 10 µM (A). Time course inhibition of Na/K-ATPase activity after 2 h, 4 h, 10 h and 24 h, at 10 µM CsA (B). C: Na/K-β1 stady-state levels after CsA treatment. Effects of different doses of CsA on Na/K-β1 protein expression levels. β-actin was used as a loading control. D–E: Effects of CsA on Na/K-β1 and CypB localization. Immunocitochemistry assays using anti-CypB and anti-Na/K-β1 rabbit polyclonal antibodies were performed on untreated (upper panel) and 10 µM CsA treated (lower panel) HK-2 cells. F–G: Effects of CsA on CypB expression. Intracellular (F) and secreted (G) CypB levels in HK-2 cells treated with different doses of CsA. Ratios between CypB and actin signals in cell lysates have been represented upon quantification. Figures are representative of at least three independent experiments.

Although it was previously described that CsA alters the activity of the pump in the kidney [12], the underlying molecular mechanisms were not elucidated. To further explore them, we observed the effects of different doses of CsA (10 nM, 100 nM, 1 µM, and 10 µM) on Na/K-ATPase activity in HK-2 cells, after 24 h treatment. Na/K-ATPase levels were quantified by determining the p-Nitrophenylphosphate (p-NPPase) activity inhibited by ouabain. Our results showed that deleterious effect of CsA on Na/K-ATPase activity was time and dose-dependent. Decline of activity was evident at 1 µM CsA with 50% of inhibition at 10 µM after 24 h of treatment (Fig. 3A and 3B). Western blot assays performed in crude lysates of CsA-treated cells showed similar Na/K-β1 levels, at any CsA concentration tested, indicating that CsA effects on pump activity were unrelated with changes on protein content (Fig. 3C). By confocal microscopy, we observed an intracellular location of Na/K-β1 in CsA-treated cells, rather than the cell membrane distribution observed in vehicle-treated cells (Fig. 3D). In these same conditions, CypB staining was less evident (Fig. 3E). Western blot assays confirm a gradual CypB diminishment in crude cell extracts (Fig. 3F) that became very prominent at 10 µM and a CypB enrichment in conditioned media (Fig. 3G) detectable at the dose of 1 µM CsA. These results indicated that the dose-dependent decrease of intracellular CypB levels after CsA treatment, correlated with altered pump activity and Na/K-β1 location.

### Na/K-ATPase location and activity in CypB silenced human kidney cells (HK-2)

**Figure 4:**
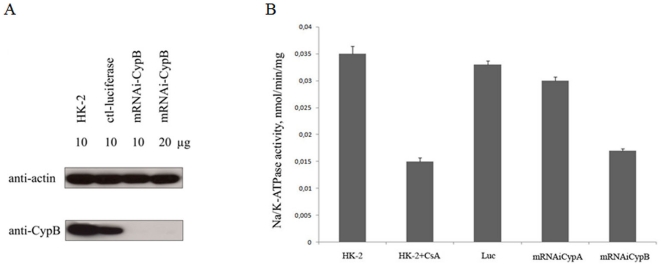
Na/K-ATPase activity decreases in CypB silenced human kidney cells (HK-2). A: HK-2 cells were silenced for CypB using microRNA-based shRNA lentiviral vectors (mRNAi-CypB). Interference of the luciferase gene was used as control (ctl-luciferase). Western blot assays show a complete lack of CypB in CypB silenced cells, even at saturating protein concentrations from cell lysates (20 µg). B: Na/K-ATPase activity decreases in silenced CypB cells in a similar manner than 10 µM CsA treatment for 24 h does in HK-2 cells. Luciferase silenced HK-2 cells were used as a control. CypA silencing in HK-2 cells does not have any effect on activity (not shown). Figures are representative of at least three independent experiments.

To further prove the involvement of CypB on Na/K-ATPase location and activity, HK-2 cells were silenced for CypB using the lentiviral system CypB-shRNA or Luc-shRNA (negative lentivirus control). Expression of CypB in silenced cells was determined by western-blot and compared with levels of actin-β, which was used as loading control. Results in Fig. 4A clearly demonstrated that CypB expression was fully inhibited in this lentiviral siRNA silenced cell system. The Na/K-ATPase activity on interfered CypB cells was similar to values observed in parental HK-2 cells treated with doses of CsA that provoked the lost of intracellular CypB. Cells silenced for CypA or luciferase showed no effects on Na/K-ATPase activity (Fig. 4B).

**Figure 5:**
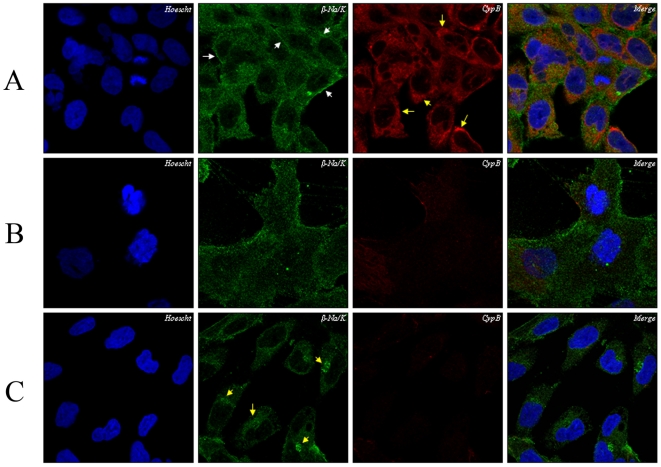
Localization of Na/K-ATPase beta and CypB in silenced or CsA-treated HK-2 cells. Immunocitochemistry assays using anti-CypB rabbit polyclonal antibody and mouse monoclonal anti-Na/K-β1 antibody were performed on luciferase silenced HK-2 control cells (ctl-luciferase) (Panel A), 10 µM CsA treated ctl-luciferase cells, for 24 h (Panel B) and Cyp B silenced cells (Panel C). White arrows point to plasma membrane and yellow arrows to endoplasmic reticulum localization. Fluorescence labelling was visualized in a Leica DM IRBE confocal microscope. Figures are representative of at least three independent experiments.

The location of endogenous Na/K-β1 subunit in CypB-shRNA silenced HK-2 cells (Fig. 5. Panel C) was observed and compared with the effects produced by CsA-treatment in Luc-shRNA HK-2 cells (Fig. 5. Panel B). In non-treated Luc-shRNA HK-2 control cells, the Na/K-β1 subunit is located in the plasma membrane (Fig. 5. Panel A, white arrows) and the cytosol, following a pattern compatible with the ER, as shown by CypB distribution (Fig. 5. Panel A, yellow arrows). In the presence of CsA, the Na/K-β1 subunit shows a diffused location (Fig. 5. Panel B), which is in accordance with effects in parental non-modified HK-2 cells treated with CsA (See Fig. 3. Panels D–G). In CypB-shRNA silenced HK-2 cells, Na/K-β1 remains at the ER and is not found in the plasma membrane (Fig. 5. Panel C, yellow arrows). In conclusion, CsA treatment or CypB silencing impair Na/K pump targeting to the plasma membrane.

### Na/K pump subunits distribution upon subcellular fractionation of CsA-treated or CypB-silenced HK-2 cells

**Figure 6:**
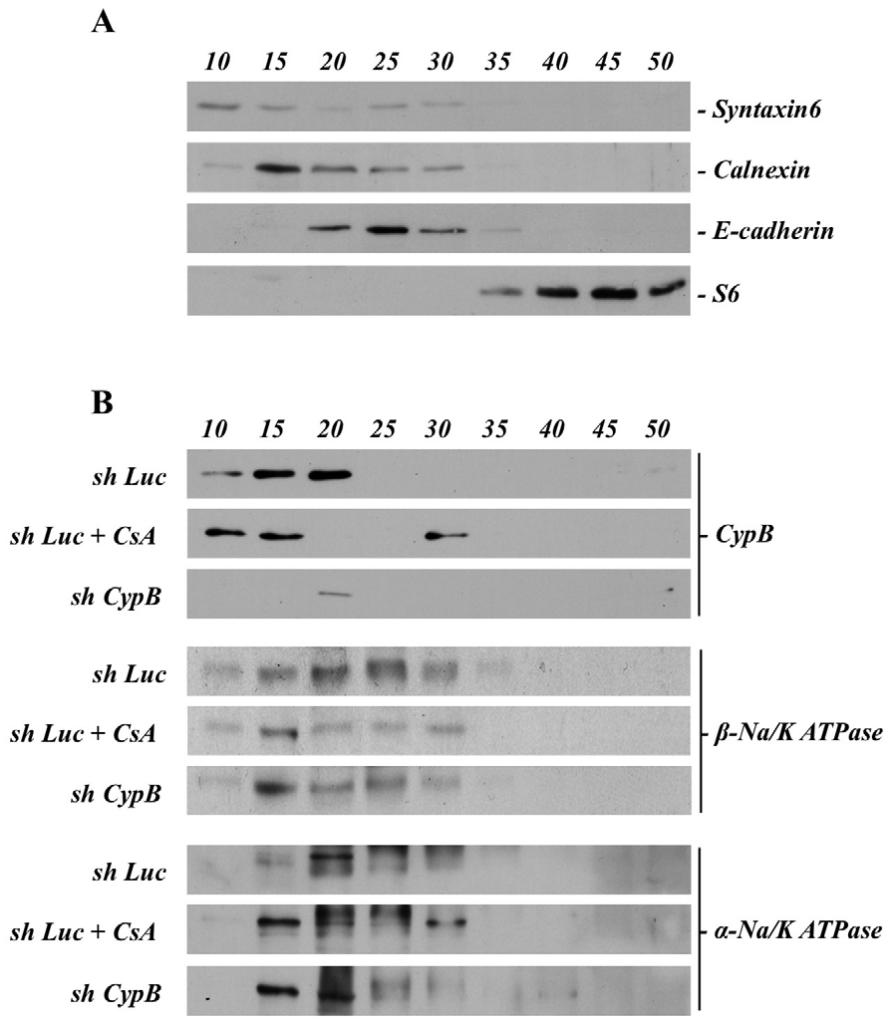
Na/K pump subunits distribution upon subcellular fractionation of CsA-treated or CypB-silenced HK-2 cells. Sh Luc, Sh Luc treated with CsA, and Sh CypB cells show different patterns of sedimentation on sucrose gradients of α and β Na/K ATPase and CypB. Cells treated or untreated with CsA were harvested and loaded on sucrose gradients (10 to 50% w/v) as described in Methods. (A) Western Blot of fractions eluted from sucrose gradient of Sh Luc cells probed against protein markers of specific cell compartments: Syntaxin-6 (Golgi), Calnexin (ER), E-cadherin (Plasmatic Membrane) and S6 (Ribosomes). (B) Western Blot of fractions eluted from sucrose gradient of Sh Luc, Sh Luc + CsA, and Sh CypB cells probed against α and β Na/K ATPase and CypB. Figures are representative of at least three independent experiments.

To further assess the distribution of beta and alpha subunits in CsA-treated or CypB silenced HK-2 cells, subcellular fraccionation followed by western blot assays was performed. Results shown in Fig. 6A indicate the distribution of representative subcellular markers along a sucrose gradient (from 10% to 50% sucrose concentration), obtained upon fractionation of Luc-shRNA HK-2 nuclei-free cell extracts. As shown, the Golgi marker syntaxin 6 is mainly present in the 10% fraction; the ER marker calnexin is more prominent in the 15% fraction; the plasma membrane marker E-cadherin is mainly present in the 25% fraction, and the ribosomal marker S6 more prominent at the heavier densities.

In those same samples, we observed that in control cells (sh Luc) CypB is predominantly found in the 15% and 20% fractions. After CsA treatment (sh Luc+CsA), CypB disappears from the 20% fraction and appears in the 30% fraction. In CypB silenced cells (sh CypB) the residual CypB is only present in the 20% fraction (Fig. 6B). To correlate the location of CypB with pump subunits, these same membranes were rehybridized with specific antibodies against alpha and beta subunits. In non-treated cells, the beta subunit is mainly distributed into the 15%, 20%, 25% and 30% fractions. Upon CsA treatment, and also after CypB silencing, the Na/K-β1 diminishes in the 20%, 25% and 30% fractions in relation to the 15% fraction that appears relatively enriched (Fig. 6B), following the same pattern than the ER marker calnexin. The alpha subunit is mainly located in the 20% fraction in control cells. After CsA treatment, Na/K-α1 shifts from the 20% to the 15% and 30% fractions, paralleling CypB distribution under this treatment. In CypB silenced cells, the alpha subunit follows a similar location than the beta, remains in the 20% fraction and becomes enriched in the 15% fraction (Fig. 6B).

## Discussion

Cyclophilins, first discovered as the intracellular receptors of the immunossupressant CsA, facilitate protein folding via their PPIase activity and contribute to the maturation of several proteins [13], [14]. A second but potentially more important role for cyclophilins is as chaperone for protein trafficking and macromolecular assembly. Among cyclophilins, CypB is targeted to the secretory pathway via an ER signal sequence. CypB accumulates both in the ER and in complexes on the plasma membrane and is specifically mobilized by CsA that promotes its secretion into the medium [15]. It was postulated a role for CypB as a chaperone to proteins destined for the plasma membrane, rather than solely as a proline isomerase functioning within the ER. This concept has been further reinforced, since CypB has been found to be part of the ER chaperone complex [16] and involved in protection against ER stress [17].

In this report, we focused towards the identification of novel CypB-binding proteins in kidney, in order to: i) learn more about the endogenous functions of CypB and ii) unravel, in part, the molecular mechanisms of CsA-induced kidney toxicity. We identified an interaction between CypB and Na/K-β1 in human kidney that was further confirmed in the proximal tubule derived HK-2 cells, and in Na/K-β1 transfected COS-7 cells. Confocal microscopy images revealed that the ER is the site for CypB and Na/K-β1 transient interaction. Na/K-ATPase is an integral basolateral membrane protein that participates in the maintenance of cell volume and electrochemical gradients [18]. The functional Na/K-ATPase is a heterodimeric ion pump that consists of one α and one β subunit. The α subunit comprises the catalytic and transport activities of the Na/K-ATPase, while the β subunit is involved in the structural maturation and correct routing of the functional heterodimeric Na/K-ATPase to the plasma membrane [19]. In agreement with this, we have also found the β-interacting α subunit in the CypB immunoprecipitates.

It is known that CsA inhibits Na/K-ATPase activity. This was first described in rat kidney cortical homogenates [20] and in rat nephron specific-segments [12]. Clearance studies showed that intraperitoneal administration of CsA for one week causes a distal tubular acidification defect in rats [5] and a significant reduction in sodium and potassium excretion [6]. CsA-treated rats also have an impaired ability to excrete a potassium load, despite serum potassium concentrations that exceed 8.4 mEq/liter. CsA interferes with potassium homeostasis in humans as well [21]. CsA-induced hyperkalemia and decreased kaliuresis could be a consequence of impaired potassium secretion and ultimately arising from CsA-induced inhibition of pump activity in potassium secreting nephron segments. Moreover, inhibition of Na/K-ATPase by CsA is very relevant in the kidney since export of sodium from the cells provides the driving force for several facilitated transporters which are important for transepithelial transport and because translocation of sodium from one side of the epithelium to the other creates an osmotic gradient that drives absorption of water, which is crucial for blood pressure control [22].

Albeit its relevance for kidney function, the exact molecular mechanisms involved on the inhibition of the Na/K-ATPase by CsA are not completely elucidated. In the rat nephron, it was reported that CsA at 0.5 µM for 30 minutes inhibited Na/K-ATPase activity in cortical collecting ducts (CCD), medullary thick ascending limbs (mTAL) and outer medullary collecting ducts from the outer stripe (OMCD0), by 35%, 53%, and 39%, respectively [12]. Calcineurin involvement was later suggested since FK506 and two other calcineurin inhibitors, significantly reduced Na/K-ATPase activity in these nephron segments [23]. Remarkably, under these conditions (and up to 2 µM CsA for 60 minutes) Na/K-ATPase activity did not change in proximal tubule S1, S2, and S3 subsegments [12], [23]. Our results are in accordance with these data since the proximal tubule derived HK-2 cells required higher doses of CsA and longer treatment times for maximal pump activity inhibition. Although it would be very interesting to evaluate the effects of CsA in Na/K-ATPase translocation and activity in the tubular distal cells as well, in order to correlate with the hypercalemia induced by CsA treatment in vivo, few cell lineages originated in human kidney exist and, to our knowledge, no cell lines of distal origen are currently available. Additionally, it has been reported that distal epithelial cells from human kidney undergo transdifferentiation to a more proximal phenotype in culture [24].

Since maximal pump impairment by CsA correlated with a concomitant intracellular CypB diminishment (about 5-fold. See Figure 3F) at doses much higher (10 µM) than the ones required for calcineurin inhibition (at the nM range), we reasoned that impairment of the pump relates with the drop of intracellular CypB levels. The fact that CypB silencing, where calcineurin is not inhibited, also reduces the Na/K-ATPase activity supports the concept that in proximal tubule segments, CypB represents a key molecule for pump function.

Deletereous effects of CsA on Na/K-ATPase activity have been mainly focused on post-translational mechanisms involving the catalytic alpha subunit [25], [26]. Phosphorylation of the alfa subunit by PKC activation which can be, in turn, promoted by CsA [27], represents the triggering signal for pump endocytosis into endosomes via a clathrin vesicle-dependent mechanism [28]. To the best of our knowledge, there is no data concerning the effects of CsA on the regulatory Na/K-β1 subunit. It is well recognized that the regulatory beta subunit acts as a chaperone that allows the folding of newly synthetized alpha subunits, directs their targeting from the ER to the appropriate plasma membrane domain and stabilizes them within the membrane, being an absolute requirement for pump and ATPase activities [29]–[31]. In this report, we describe for the first time a novel interaction between CypB and Na/K-β1 and demonstrate that CypB is required for pump activity in proximal tubule cells. We showed that CypB silencing produced similar effects on Na/K-ATPase activity than CsA treatment resulting in enrichment of both alpha and beta subunits in the ER, what suggests a failure on the maturation and routing of the pump from this compartment towards the plasma membrane. These data indicate that CypB through its interaction with Na/K-β1 might regulate maturation and trafficking of the pump through the secretory pathway. A similar mechanism has been described for the Na/Kα1-ATPase subunit and the scaffold ankyrin cytosolic protein. The presence of an ankyrin-binding sequence in Na/Kα1-ATPase subunit facilitates its transport in the secretory pathway in Madin-Darby canine kidney cells and in other cultured cells [32].

According to the correlation observed between CsA doses, intracellular CypB levels and pump activity, we propose that the deleterious effects of CsA on tubular function have to do, at least in part, with CypB secretion promotion. The involvement of CypB in human disease has only been recently unravealed. For instance, it has been described that CypB forms a complex with CRTAP (cartilage-associated protein) and prolyl 3-hydroxylase 1 and that mutations on any of these three proteins cause recessive form of esteogenesis imperfecta and loss or decrease of type I collagen prolyl 3-hydroxylation [33]–[35], which is a mechanism for connective tissue disease. Expression of CypB has been associated with malignant progression and regulation of genes implicated in the progression of breast cancer [36]. CypB has also been recognized as a partner of CD147. CD147 has been shown to function as a signalling receptor for Cyclophilin A and B to mediate chemotactic activity of cyclophilins towards a variety of immune cells [37]. More recently, it has been reported that agents targeting either CD147 or cyclophilin activity showed significant antiinflamtory effects, suggesting that CD147-cyclophilin interactions may be a good target for new anti-inflammatory therapeutics [38].

In the present report we claim an essential and novel role for CypB in the maturation and trafficking of Na/Kα1-ATPase pump through the secretory pathway. A series of important studies have demonstrated non-pumping functions for the Na/K-ATPase that include regulation of oncogenic transformation [39], epithelial to mesenchymal cell transition [40], tight junction formation [41] and control of the plasma membrane cholesterol distribution [42]. The novel interaction found between CypB and Na/K-β1 and the importance of CypB as a Na/K-ATPase functional modulator should be very relevant to further understand the role of CypB in renal and extrarenal pathophysiology.

## Materials and Methods

### Cell lines, cultures and reagents

The proximal tubule epithelial derived cell line HK-2 (CRL-2190) (immortalized by transduction with HPV-16) [43] was obtained from the American Type Culture Collection (Manassas, VA) and was cultured in Dulbecco's modified Eagle's medium (DMEM)-Ham's F12 (1∶1 v/v); 60 nM sodium selenate; 5 µg/ml transferrin; 2 mM glutamine; 5 µg/ml insulin; 50 nM dexamethasone; 5 nM triiodothyronine; 10 ng/ml epidermal growth factor; 20 mM D-glucose; 2% fetal calf serum; 20 mM HEPES, pH 7.4]. The COS-7 cell line (American Type Culture Collection, Manassas, VA), an African green monkey kidney fibroblast-like derived cell line, was maintained in DMEM supplemented with 10% fetal bovine serum (FBS), 1% L-glutamine, 1% nonessential amino acids, penicillin (100 U/mL), and streptomycin (100 U/mL) in 5% CO2. Media for cell culture and supplements were obtained from Biological Industries (Kibbutz Beit, Haemek, Israel). For CsA treatment, HK-2 cells were cultured into 100 mm plates up to confluence. CsA (Calbiochem) was added to the plates at different doses (0 nM, 10 nM, 100 nM, 1 µM, 10 µM, 20 µM) at different times. The CsA doses used in this study have been previously utilized for us and others to obtain a nephrotoxic profile in vitro that would be relevant to the clinical situation in patients, based on the CsA concentration range reached in kidney tissue [44]–[46]. Cells lysates and conditioned media from each situation were collected and stored for immunoblotting, as described [44].

### Yeast Two-Hybrid Assays

A yeast two-hybrid screening was performed using a human kidney MATCHMAKER cDNA Library (BD Biosciences Clontech. Palo Alto, CA). The complete human CypB (hCypB) cDNA sequence was used as bait. The library was co-transformed with the pGBKT7-GAL4 containing CypB, into AH109 competent yeast cells following the manufacturer's instructions. Positive and negative controls provided with the library were used. As a positive control, the pGBKT7-53 that corresponds to the fusion of GAL4 binding domain with p53 protein and the pGADT7-T that expresses the GAL4 activating domain fused to the T antigen were used. As a negative control, the vector pGBKT7-lam, containing lamin C was used together with pGADT7-T.

### Reverse transcription-PCR

For yeast two-hybrid assays, the hCypB cDNA was obtained from fresh kidney surgical samples, corresponding to the normal counterpart tissues of patients bearing a renal cancer tumor. Samples were obtained at the time of surgery upon approval by the Institutional Review Board, and informed consent from patients before surgery. Total RNA was isolated using guanidine thiocyanate as described elsewhere [47]. For cDNA synthesis and PCR amplification, 0.5 µg of DNAse I treated total RNA was reverse-transcribed and amplified using the Super Script One-Step reverse transcription RT-PCR System (Invitrogen Life Technologies, Calsbard, Ca, USA) in a single step according to the manufacturer's instructions.

### Plasmids

hCypB cDNA (GenBankTM accession number NM_000942) was obtained by RT-PCR using specific primers: upper 5′-CATATGATGAAGGTGCTCCTTGCC-3′, where the underlined sequence corresponds to the NdeI restriction site followed by hCypB cDNA sequence at positions 1 to 18 from the translation initiation site, and lower 5′ CTGCACAAAGATGTCCCTGTGCCCTA-3′, where underlined sequence corresponds to PstI site and the rest to hCypB cDNA sequence, from positions 625 to 644 to the translation initiation site, which corresponds to positon 194 of the GenBank sequence. The cDNA was cloned into the NdeI-PstI restriction endonuclease site of pGBKT7-GAL4 (Clontech) and used in yest two-hybrid assays, as stated above. The pGEX-6P1-hCypB expression vector was constructed by PCR amplification of hCypB cDNA using specific primers (5′GGATCCGCCGATGAGAAGAAGAAGGG-3′ and 5′CCCGGGAAAGATGTCCCTGTGCCCTA-3′) and ligated into the BamHI-SmaI restriction sites of pGEX-6P1 (Amersham) for further expression and purification of recombinant GST-hCypB fusion protein. The Na/K-β1 subunit, obtained from the yeast two-hybrid library pray clone (in PCAT. BD Biosciences, clontech, Palo Alto, CA), was subcloned into the BglII restriction endonuclease site of pCMV-HA mammalian expression vector (BD Biosciences, clontech, Palo Alto, CA) to get the pCMV- Na/K-β1 -HA construct.

### Expression and Purification of Recombinant Proteins

BL21 bacterial cells were transformed with constructs expressing GST and GST-hCypB proteins. Luria broth (500 ml) containing 50 µg/ml ampicillin was inoculated with 5 ml of bacteria and incubated in an orbital shaker at 37°C. Bacteria were grown to A600 = 0.3, induced with 0.5 mM isopropyl-1-thio-β-D-galactopyranoside, and shaken for 5 h at 37°C. The GST, and GST-hCypB soluble proteins, obtained upon sonication, were purified following the protocol for GSTrap FF 1 ml columns (Amersham Biosciences). Protein concentration was not measured. The expression level of the recombinant protein was evaluated in comparison with endogenous proteins after electrophoresis on 10% polyacrylamide denaturing gels stained with Comassie Blue.

### Transfections assays

Monolayers of COS-7 cells grown in 70% confluence were transfected with 5 µg of pCMV-HA-Na/K-β using LipofectAMINE (Life Technologies Ltd.) according to the manufacturer's instructions. 24–48 h after transfection, cells were trypsinized. After transfection, cells were lysed with a buffer containing 20 mM Tris, 200 mM NaCl, 1 mM EDTA, 0.5 NP-40 and Protease inhibitor Cocktail (SIGMA).

### GST pull-down and co-immunoprecipitation assays

10 µg of GST and GST-CypB purified proteins (see above) were incubated with 30 µl of Glutathione Sepharose 4B (GE Healthcare) and 400 µl of enriched HA-Na/K-β1cell lysates (see above), overnight at 4°C. Next day, beads were washed carefully with lysis buffer three times. Then the beads were pelleted, mixed with 15 µl SDS-sample dye mix, boiled for 5 min and subjected to immunoblot analysis using specific anti-Na/K-β1 rabbit polyclonal antibody (H115: sc-25709, Santa Cruz biotechnology).

HK-2 cells were cultured into 100 mm plates up to confluence, harvested and lysed with a buffer containing 20 mM Tris, 200 mM NaCl, 1 mM EDTA, 0.5 NP-40 and Protease inhibitor Cocktail (SIGMA). Cell lysates were incubated overnight with anti-CypB (PA1-027, Affinity Bioreagents), anti-Na/K-β1 (M17-P5-F11, Mouse mAb #ab2873 ABCAM) and anti-Na/Kα1 (M8-P1-A3, Mouse mAb #ab2872 ABCAM) primary antibodies, respectively. ChromePure rabbit IgG and ChromePure mouse IgG (Jackson ImmunoResearch) were used as control. Next day, 30 µl of protein G (SIGMA) was added into each tube and incubated for 1 h at 4°C. The beads were washed three times with lysis buffer and solubilized in 15 µl SDS sample buffer.

### Western-Blotting

Samples were analyzed by SDS-12% polyacrylamide gel electrophoresis (SDS-PAGE) under reducing conditions. Proteins were transferred to a PVDF membrane (Schleicher&Schuell). Blots were blocked for 1 h in PBS containing 5% non-dried milk and incubated with the appropiate primary antibodies in the same blocking buffer for 2 h at room temperature, anti-Na/K-β1 1∶1000 (ABCAM), anti-Na/Kα1 1∶1000 (See above) and 1∶2000 anti cyclophilin B (See above). When indicated, anti-actin rabbit polyclonal antibody (A5060, SIGMA) was included for protein loading control. After incubation, blots were extensively washed and incubated with horseradish peroxidase-conjugated mouse anti-rabbit and anti-mouse secondary antibodies for 1 h at room temperature. After washing, sensitive detection of bound antibody was carried out using Amersham ECL Plus Western Blotting (GE healthcare, UK), manufacturer's protocol was applied.

### Sucrose Gradient

HK-2 cells were harvested with 25 mM MES pH 6.5 buffer containing 1% triton X-100 and protease inhibitors and homogenized with a teflon Dounce homogenizer. The lysates were centrifuged at 2000 rpm to pellet the nuclei and the postnuclear supernatant was layered on the top of a discontinuous 10–50% (w/v) sucrose gradient (total volume 11.4 mL) made in 25 mM MES buffer (pH 6.5) containing 150 mM NaCl. Gradients were centrifuged 16 h at 260000 rcf on a Sorvall TH-641 rotor. Nine fractions corresponding to 10, 15, 20, 25, 30, 35, 40, 45 and 50% (w/v) sucrose were collected from the top of the gradient, precipitated with TCA and washed with acetone. Pellets were resuspended in Laemmli Buffer and subjected to western blot analysis as described earlier. Specific anitibodies against Syntaxin6 1∶1000 (Mouse mAb #S55420 Transduction Labs), Calnexin 1∶1000 (Rabbit Polyclonal #SPA-860 STRESSGEN), E-cadherin 1∶2500 (Mouse #610181 BD Biosciences) and S6 ribosomal protein 1∶1000 (5G10) Rabbit mAb #2217 Cell Signaling) were used for identification of subcellular cell compartments in sucrose gradient fractions.

### Immunocytochemistry

HK-2 cells were allowed to spread on microscope cover glasses (Marlenfeld GmbH & Co.KG) for 48 h, and further treated with CsA (10 µM) for 24 h. Immunofluorescence staining of CypB and Na/K-β1 was performed by washing slides twice in cold PBS and fixed in cold 4% paraformaldehyde for 1 h followed by three 10-min PBS washes. Aldehyde groups were blocked with 50 mM NH2Cl for 30 min and washed for 5 min in PBS three times. Cells were permeabilized with 0.1% triton X-100 for 10 min, followed by washing with PBS twice and further blocking with 1% BSA in PBS for 30 min. Slides were incubated for 90 min at room temperature with 1∶100 anti anti-Na/K-β1 rabbit polyclonal antibody (Fig. 3) (H115: sc-25709, Santa Cruz biotechnology) or with 1∶100 anti-Na/K-β1 mouse monoclonal antibody (Fig. 2 and 5) (ABCAM) and 1∶100 anti cyclophilin B rabbit polyclonal antibody (PA1-027, Affinity Bioreagents). After washing, cells were incubated with secondary antibodies (1∶300 Anti-rabbit-TRITC, T 5268, SIGMA; 1∶500 Goat anti mouse Alexa Fluor 488 [A11029] and 1∶500 Goat anti rabbit Alexa Fluor 568 [A11036]). Slides were washed thrice with PBS followed by nuclei staining with Hoechst 33342 (Sigma). Fluorescence labelling was visualized in a confocal spectral FV 1000 Olympus microscope.

### Na/K-ATPase activity assay

Na+-K+-ATPase activity was estimated on the basis of measurement of p- nitrophenylphosphate (pNPP) hydrolysis. Protein extracts from cells treated with 0 nM, 10 nM, 100 nM, 1 µM and 10 µM CsA for a 24 h period, or treated with 10 µM CsA for 2 h, 4 h, 8 h, 10 h and 24 h, were incubated with 0.5 ml of the buffer (50 mM Tris, 10 mM MgSO4, 5 mM EDTA, 90 mM KCl, pH 7.6) at 37°C for 30 minutes. Then, 0.5 ml of buffer containing 12 mM p-nitrophenylphosphate with or without ouabain were added to each sample. The samples were incubated 30 minutes at 37°C, the reaction was stopped by adding trichloroacetic acid 30%. After for 5 min at 13200 rpm, supernatant was collected and added into a spectrophotometer cuvette containing 2 ml Tris 1 M, each sample was read at 410 nm absorbance.

### shRNA transfections

MicroRNA-based shRNA lentiviral vectors were produced by co-transfecting 293FT cells with transfer vectors encoding the puromycin resistance gene and a shRNA targeting CypB or a control non-targeting shRNA, the HIV-1 packaging plasmid psPAX2, and a VSVg expression plasmid (pMD2.G) using calcium precipitation. The transfection medium was exchanged the following day and viral supernatant was harvested 48 hrs after transfection, clarified by centrifugation (5 min at 200× g), and filtered through a 0.45 mm syringe filter (Sarstedt). Kidney cells were transduced and placed in selection medium containing 5 mg/ml puromycin (Sigma) 72 hours after transduction. shRNA targeting sequences: control non-targeting shRNA: ctctcgcttgggcgagagtaag. The hairpin sequence used for CypB was; gccgggtgatctttggtctctt, which encompases nucleotides 149 to 170 from the translation initiation site of the hCypB sequence.
